# Adult-Onset Neuronal Ceroid Lipofuscinosis With a Novel *DNAJC5* Mutation Exhibits Aberrant Protein Palmitoylation

**DOI:** 10.3389/fnagi.2022.829573

**Published:** 2022-04-08

**Authors:** Qiang Huang, Yong-Fang Zhang, Lin-Jie Li, Eric B. Dammer, Yong-Bo Hu, Xin-Yi Xie, Ran Tang, Jian-Ping Li, Jin-Tao Wang, Xiang-Qian Che, Gang Wang, Ru-Jing Ren

**Affiliations:** ^1^Department of Neurology, Institute of Neurology, Ruijin Hospital, Shanghai Jiao Tong University School of Medicine, Shanghai, China; ^2^Department of Pharmacology and Chemical Biology, Shanghai Jiao Tong University School of Medicine, Shanghai, China; ^3^Shanghai Collaborative Innovation Center for Translational Medicine, Shanghai Jiao Tong University School of Medicine, Shanghai, China; ^4^iHuman Institute, Shanghai Tech University, Shanghai, China; ^5^Department of Biochemistry, Center for Neurodegenerative Disease, Emory University School of Medicine, Atlanta, GA, United States; ^6^Department of Neurology, Shanghai East Hospital, Tongji University School of Medicine, Shanghai, China

**Keywords:** cysteine string protein α, adult-onset neuronal ceroid lipofuscinosis, neurodegenerative disease, lysosome, cognitive decline

## Abstract

Neuronal ceroid lipofuscinosis (NCL) is composed of a group of inherited neurodegenerative diseases, with the hallmark of lipofuscin deposit (a mixture of lipids and proteins with metal materials) inside the lysosomal lumen, which typically emits auto-fluorescence. Adult-onset NCL (ANCL) has been reported to be associated with a mutation in the *DNAJC5* gene, including L115R, L116Δ, and the recently identified C124_C133dup mutation. In this study, we reported a novel C128Y mutation in a young Chinese female with ANCL, and this novel mutation caused abnormal palmitoylation and triggered lipofuscin deposits.

## Introduction

Neuronal ceroid lipofuscinosis (NCL) is a group of inherited neurodegenerative diseases and is typified by the abnormal accumulation of lipofuscin (a mixture of lipids, proteins, and metal materials) inside the lysosomal compartment, which emits auto-fluorescence (Haltia and Goebel, 2013; Mole et al., 2019). Despite significant clinical variability, common symptoms include cognitive decline, motor dysfunction, seizures, visual impairment, and even premature death due to prominent neurodegeneration. The NCLs are classified according to the age at onset, and there are at least 13 distinct genes identified in different patients, including *CLN1/PPT1*, *CLN2/TPP1*, *CLN3*, *CLN4/DNAJC5*, and *CLN14/KCDT7* (Naseri et al., 2021) (NCL mutation and patient database)^1^. Therapies, such as gene therapy and stem cell therapy, are promising for NCL, though currently remaining at the early stage (Mole et al., 2019; Naseri et al., 2021).

Most pathogenic genes of NCL harbor the autosomal recessive inheritance, while CLN4/*DNAJC5* is exceptional and results in the autosomal dominant adult-onset NCL (AD-ANCL). Patients with AD-ANCL developed their symptoms during adulthood. One research summarized the symptoms of the reported case series of AD-ANCL (45 patients), with most patients developing seizures at around 25, 8.8% patients presenting with memory loss, and 20% patients with motor dysfunction (Naseri et al., 2021). A heterozygous point mutation (p.L115R) and an in-frame codon deletion (p.L116Δ) in the *DNAJC5* gene have been identified in pedigrees of AD-ANCL. Most research underlying the mechanism of AD-ANCL focused on the L115R and L116Δ mutations, which limits the understanding of the disease itself. In a recent study, a 30-bp duplication in the *DNAJC5* gene (p.C124_C133dup) has been identified in two brothers, whose mother died from AD-ANCL at the age of 56 (Jedličková et al., 2020), indicating the association between *DNAJC5* genotype and clinical phenotype.

DnaJ heat shock protein family (Hsp40) member C5 (*DNAJC5*) encodes cysteine string protein α (CSP α), a protein of 198 amino acids (around 22 kDa). The CSP α is located at the synapses and is vital for synaptic function (Burgoyne and Morgan, 2015; Nieto-González et al., 2019; Gundersen, 2020). The CSP α targets the organelle membrane to mediate neurotransmitter release and membrane fusion. Deletion of the CSP α leads to a decrease in SNAP-25 and the downstream soluble NSF attachment receptor (SNARE) complex assembly (Chandra et al., 2005; Sambri et al., 2017). Homozygosis knock-out of the CSP α in the mouse model induced the significant neurodegeneration (Fernández-Chacón et al., 2004).

In this study, we reported a case of a Chinese female carrying the novel p.C128Y mutation in the *DNAJC5* gene and further verified its pathogenicity *in vitro* and *in vivo*.

## Materials and Methods

### Human Subject Research

All procedures were approved by the ethical committee of Ruijin Hospital, Shanghai Jiao Tong University School of Medicine. Informed consent was obtained from the participant and her parents. The DNA was extracted from blood samples using the QIAamp DNA Mini Kit (Qiagen, Germany). Whole-exon sequencing was applied to identify the genetic profile of the patient, and the detected mutation was further verified by the PCR. Brain MRI was performed on a 3.0 T scanner (Philips, United States). Around 3-mm deep punch of medial upper arm was taken for observing the sweat glands under transmission electron microscopy (TEM).

### *In silico* Analysis

The substitution score for Cys128 was quantified by the BLOSUM62 matrix^2^. The potential impacts of L115R and C128Y mutation on the function and structure of the CSP α were assessed by Mutation Taster^3^ PolyPhen-2^4^, SIFT, and PROVEAN^5^. The influence of amino acid substitution on the palmitoylation potential was evaluated using CSS-Palm 4.0. Furthermore, the hydrophobicity of the cysteine-string domain was assessed using the Kyte-Doolittle algorithm^6^.

### Plasmid and Lentivirus Construction

To build plasmid overexpressing wild-type (WT) CSP α, the *DNAJC5* cDNA (NM_025219) was inserted into the PGEX-KG vector between *Eco*RI and *Xba*I restriction sites. Based on the WT plasmid, site-directed mutagenesis was performed using the QuickChange™ site-directed mutagenesis kit (Stratagene, United States) to construct L115R and C128Y mutation according to the manufacturer’s instruction. The primers for the plasmid verification were in the Supplementary Table 1. To build the lentiviral vector accordingly, human cDNA encoding wild-type CSP α and C128Y mutation were cloned into a Lenti-GFP vector. The viral particles were concentrated by ultracentrifugation at 20,000 rpm for 120 min at 4°C using a Beckman XPN-100 ultracentrifuge.

### Cell Culture and Cell Transfection

The SH-SY5Y cells were maintained in Dulbecco’s Modified Eagle Medium (DMEM), supplemented with 10% fetal bovine serum (FBS) and penicillin-streptomycin (Gibco, United States) at 37°C in a 5% CO_2_ incubator. One day before transfection, the cells were cultured in a 6-well plate at a density of 0.3 × 10^6^ cells/well. Cell transfection was performed with C128Y, L115R, WT CSP α plasmid, or vector using the Lipofectamine 3000 (Thermo Fisher Scientific, United States) according to the manufacturer’s instruction. Forty-eight hours after transfection, the cells were harvested for downstream analysis.

### Cell Viability

Cell viability after transfection was quantified using the CellTiter-Lumi™ plus assay (Beyotime, China) according to the manufacturer’s instructions. Forty-eight hours after transfection, 100 μl reagent was added to each of the parallel wells for 10-min incubation. The plates were read using standard luminescence settings on a Synergy MX plate reader (BioTeck, United States).

### Animal and Stereotactic Injection

The C57BL/6J mice (female, 5 months old, 3 for each group) were purchased from the GemPharmatech company. The animals were kept in a specific pathogen free (SPF) environment with a 12:12 light-dark cycle with free access to food and water. Animal experiments were performed according to the NIH Guide for the Care and Use of Laboratory animal. All procedures were approved by the ethical committee of Ruijin Hospital, Shanghai Jiao Tong University School of Medicine.

Isoflurane was deployed for anesthesia, and a stereotaxic instrument (RWD life science, China) was used to locate the hippocampal CA3 region (coordinates: –2.8 mm anteroposterior, ± 20.5 mm mediolateral, and –3 mm dorsoventral). Before injections, lentiviral vectors were diluted with sterile phosphate-buffered saline (PBS) to achieve a titer of 1 × 10^8^ TU/ml and shortly stored on ice. Each mouse was transcranially injected with 2 μl Lvv-GFP-CSP α^WT^, Lvv-GFP-CSP α^C128Y^, or Lvv-GFP (vector) bilaterally using a 10-μl syringe over 5 min. The needle remained in place for 5 min after complete injection and then was slowly removed. Two months after stereotactic injection, the mice were sacrificed. One hemisphere was fixed in 4% paraformaldehyde (PFA), and the other hemisphere was fixed in the 2.5% glutaraldehyde.

### Histochemistry Staining

Fixed brain tissue was embedded with paraffin, sectioned, and mounted on slides. The tissue section then underwent procedures of deparaffinization and rehydration. For periodic acid-Schiff (PAS) staining, tissues were oxidized in 0.5% periodic acid solution for 5 min and rinsed in distilled water three times. Then, the sections went for incubation sequence of Schiff’s reagent (15 min) and Mayer’s hematoxylin (1 min), with an interval of 5 min washing under tap water. For long Ziehl-Neelsen staining, the sections were incubated in the carbol-fuchsin solution overnight and followed by incubation in methylene blue solution for 1 min after washing in the tap water. After staining, the sections were incubated in the gradient alcohol for dehydration and mounted by the permanent mounting medium (VectorLab, United States). The images were captured by the Leica DM6B microscope system.

### Immunofluorescent Staining

After deparaffinization and rehydration, the sections were blocked with 5% bovine serum albumin (BSA) in PBS buffer for 1 h at room temperature and then incubated with anti-SNAP25 antibody (Abcam, United States, ab41455, 1:100) overnight at 4°C. The next day, after washing with PBS-T 3 times, the sections were incubated with Goat Anti-Rabbit IgG H&L (Alexa Fluor^®^ 594) (Abcam, United States, ab150077, 1:500) for 1 h at room temperature. Following washing with PBS-T 3 times, the sections were mounted with Antifade Mountant [with 4′,6-diamidino-2-phenylindole (DAPI)]) (Thermo Fisher Scientific, United States). The immunofluorescence images were captured using a Leica SP8 confocal microscope.

### Western Blot Analysis

The transfected cells were lysed using a Mammalian Protein Extraction Reagent with Halt Protease Inhibitor Cocktail (Thermo Fisher Scientific, United States) and then subjected to a 12,000 rpm centrifugation at 4°C for 10 min to collect the supernatant. Total protein concentration was determined using the BCA protein assay kit (Thermo Fisher Scientific, United States). Forty micrograms of proteins were loaded onto 12.5% sodium lauryl sulfate-polyacrylamide gel electrophoresis (SDS-PAGE) gels. After separation, proteins were transferred to 0.22 μm polyvinylidene fluoride (PVDF) membranes (Millipore, United States). The membranes were blocked for 2 h and then incubated with an anti-CSP antibody (Enzo life, United States, ADI-VAP-SV003-E, 1:1,000) and an anti-beta actin antibody (Sigma, United States, A5441, 1:1,000) overnight at 4°C. After washing three times with Tris-buffered saline with Tween-20 (TBST), the membranes were incubated with corresponding secondary peroxidase-conjugated antibodies (Beyotime, China, A0208 or A0216, 1:1,000). Protein bands were visualized with an ECL kit (Thermo Fisher Scientific, United States). The images were captured using an Odyssey Image Station (LI-COR, United States).

### Transmission Electron Microscopy

Rapidly excised blocks of the skin tissue and the mice hippocampal CA3 region were immediately fixed with 2.5% glutaraldehyde for 24 h at 4°C. Then, specimens were postfixed in 1% osmium tetroxide for 1 h at room temperature, dehydrated in an ethanol series, and embedded in Epon 812 resin. Ultrathin sections (70 nm) were collected on Formvar-coated slot grids using an ultramicrotome (Leica EM UC7, Germany) equipped with a diamond knife and stained with lead citrate. The ultrastructure images were recorded in a Talos L120C electron microscope (Thermo Fisher Scientific, United States) operated at an accelerating voltage of 80 kV.

### Statistical Analyses

All the analyses of the acquired pictures were conducted using ImageJ/Fiji plugin. Data were presented as mean ± SEM and analyzed by the GraphPad Prism 8.0 software. One-way ANOVA followed by the *post hoc* Dunnett’s test was applied to compare between each group. Statistical significance was defined as *P* < 0.05. Data visualization was based on R-4.1.0 (www.r-project.org).

## Results

### Clinical Features of the Patient With ANCL

**FIGURE 1:**
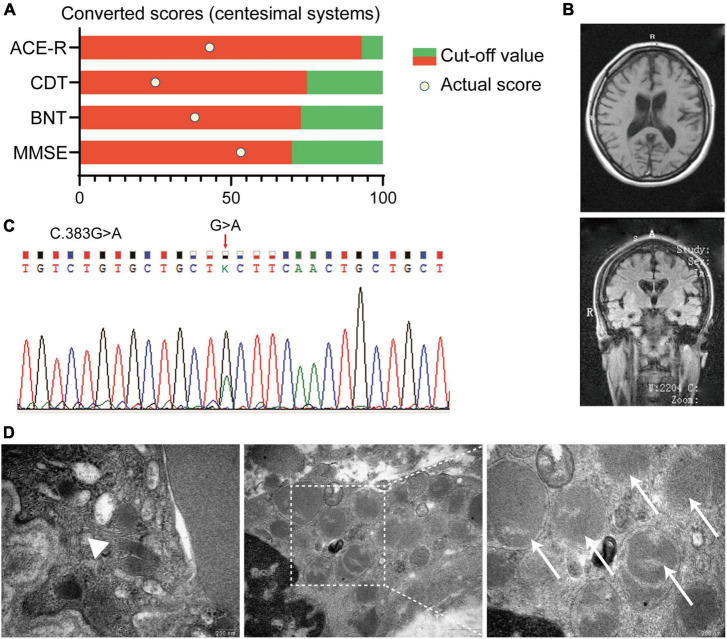
Summary of clinical records of an affected patient with Adult-onset NCL (ANCL). **(A)** Neuropsychological assessments indicated the cognitive decline in this patient. Scores of each assessment were converted to centesimal systems. **(B)** Typical MRI imaging of the proband revealed the symmetric atrophy of the bilateral cortex (GCA scale = 2) and hippocampus (MTA scale = 2). **(C)** Chromatograms of the *DNAJC5* genomic DNA sequence showed a coding variant at position c.383 (c.383G > A). **(D)** Representative pictures of the skin biopsy of sweat gland showed GRODs and curvilinear profile in the affected proband. Magnification is 65,000× in the left and right picture, and 33,000× in the middle one. White arrowhead: the curvilinear profile, white arrows: GRODs. MMSE:, mini-mental state examination; BNT, Boston naming test; CDT, clock drawing test; ACE-R, Addenbrooke’s cognitive examination; GRODs, granular osmiophilic deposits; GCA, global cortical atrophy; MTA, medial temporal lobe atrophy.

A 20-year-old female undergraduate was referred to the memory clinic, Ruijin Hospital, Shanghai Jiao Tong University School of Medicine due to progressive memory loss with personality changes reported by her parents. We reviewed her medical records, and about 9 months earlier, she paid a visit to the community hospital, and claiming memory loss. However, assessments by the primary physician were completely negative. At this visit to Ruijin Hospital, a series of neuropsychological assessments were deployed to this patient, and the result indicated the cognitive decline (Figure 1A). The patient presented with Parkinsonian motor features, including tremors in the left upper limb and bradykinesia. The specialist found her facial expression relatively decreased, while the muscle tension increased. Brain MRI revealed moderate atrophy of the bilateral cerebral cortex [Global cortical atrophy (GCA) scale = 2] and the hippocampus [Medial Temporal lobe Atrophy (MTA) scale = 2] (Figure 1B). The detailed medical records were listed in the Supplementary Material of this article.

### p.C128Y CSP α Is Associated With ANCL

**FIGURE 2:**
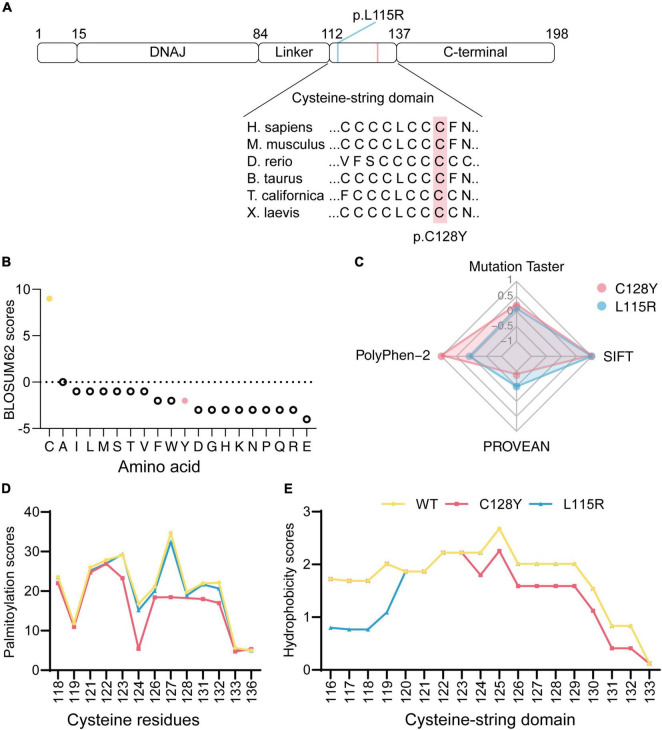
*In silico* analysis of mutated *DNAJC5*/CSP α. **(A)** Both p.L115R and p.C128Y are located at the cysteine-string domain, as visualized in the ideogram of CSP α. **(B)** The BLOSUM62 score indicated a rare substitution from cysteine to tyrosine at p.128. **(C)** Disease-causing potential, calculated with Mutation Taster, SIFT, PolyPhen-2, and PROVEAN, revealed worse pathogenicity for the C128Y mutation (1/100-fold change for scores from Mutation Taster, 1,000-fold change for scores from SIFT, 1/10-fold change for scores from PROVEAN). **(D)** The C128Y mutation decreased predicted palmitoylation score, indicating the potential difficulty of this mutant in forming disulfide bonds with palmitic acid. **(E)** C128Y mutation decreases the hydrophobicity of the cysteine-string domain.

Considering the progressive cognition decline, personality change, motor dysfunction, and noteworthy brain atrophy, we further conducted the whole-exome sequencing on this patient. A missense mutation in *DNAJC5* exon4 (C.383G>A) was identified (Figure 1C), which led to the substitution from cysteine to tyrosine at p.128 (Figure 2A). The polymerase chain reaction was deployed to verify the C128Y mutation in the proband. This mutation was not found in either of her parents. Furthermore, we did not identify any mutation of other disease-causing genes of NCL in this pedigree. Next, we performed skin biopsy and found granular osmiophilic deposits (GRODs) (Figure 1D, white arrow) and curvilinear profiles (Figure 1D, white arrowhead) by ultrastructurally using transmission electron microscopy (TEM). Taking all the clinical manifestations, neurological examination, necessary laboratory examinations (listed in the supplementary), skin biopsy, and novel C128Y mutation in *DNAJC5* gene together, the patient was eventually diagnosed as ANCL in Ruijin Hospital at the age of 20.

### *In silico* Analysis of Mutated *DNAJC5*/CSP α

The Cys128 is identified as highly conserved among many species (Figure 2A). The BLOSUM62 scores for cysteine substitution in homologous proteins were calculated (Figure 2B). A negative score resulting from cysteine to tyrosine substitution indicated rare substitution or, in other words, evolution. To predict the disease-causing potential of C128Y mutation, we included the reported L115R mutation as a positive control when using Mutation Taster, PolyPhen-2, PROVEAN, and SIFT. The C128Y mutation was predicted to induce a harmful effect on the organism but in a slightly different pattern compared to the L115R mutation (Figure 2C). Cysteine contains sulfydryl, which is an active site for s-palmitoylation with palmitic acid. Palmitoylation scores for each cysteine in mutated CSP α were calculated by CSS-Palm 4.0, and the C128Y mutation was predicted to cause a worse palmitoylation ability than L115R mutation, especially at Cys124 (Figure 2D). Considering that L115R mutation induced oligomer formation, changes of hydrophobicity after amino acid substitution were further predicted. The overall hydrophobicity of both mutant CSP α was predicted to decrease in a different pattern, and L115R mutation tended to be worse (Figure 2E).

### C128Y Mutation Caused Abnormal Palmitoylation of CSP α and Aggregates Formation *in vitro*

**FIGURE 3:**
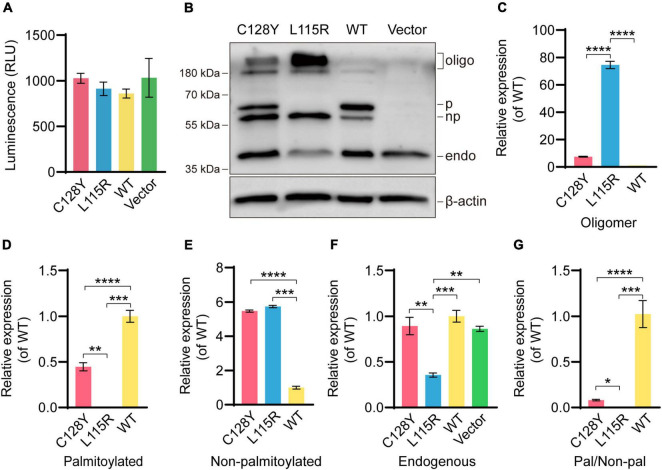
Abnormal palmitoylation and oligomer formation due to C128Y mutation *in vitro*. **(A)** Both the C128Y and L115R mutations had no impact on cell viability. **(B)** The anti-CSP α antibody (Enzo life, ADI-VAP-SV003-E) used in this study can detect the band at around ∼34 kDa, the molecular weight for endogenous CSP α. The EGFP-tagged CSP α consisted of the amino acids of both EGFP (around 26 kDa) and CSP α and exhibited an apparent molecular weight of around 60 kDa. The palmitoylation process induced a shift in gel migration of approximately 7 kDa (67 kDa is the apparent molecular weight of the palmitoylated species). Furthermore, bands above 180 kDa represent oligomers of CSP α. The L115R mutation reduced the palmitoylation process and also enhanced the amount of oligomer formation. Similarly, but to a lesser extent, the C128Y mutation-induced decreased palmitoylation and triggered oligomer formation, but without impacting abundance detected in endogenous CSP α bands, as quantified in panel **C** (oligomer), **(D)** (palmitoylated CSP α), **(E)** (non- palmitoylated CSP α), **(F)** (endogenous CSP α), and **(G)** (ratio of palmitoylated CSP α over non- palmitoylated CSP α). *N* = 3, all data in the figure are shown as mean ± SEM, each group vs. WT. *P < 0.05, **P < 0.01, ***P < 0.001, and ****P < 0.0001.

*In vitro*, neither C128Y nor L115R mutation had an impact on the cell viability after transient transfection (Figure 3A). The transiently transfected neuroblastoma cell lysates were separated by the SDS-PAGE gels, and several bands were detected by immunoblotting. As we expressed (EGFP)-tagged CSP α intracellularly, the exogenous CSP α was different from the endogenous one due to the existence of enhanced green fluorescent protein (EGFP), which provided an extra molecular weight of about 26 kDa. As reported, the palmitoylation process induces a heavier shift on migration for approximately 7 kDa. The novel C128Y mutation caused different presentations of the palmitoylation process and oligomer formation on SDS-PAGE gels compared to the wild-type (WT) CSP α, which was not similar to that from L115R mutation. The C128Y mutation caused the obstacle in the palmitoylation process, leaving a relatively higher portion of protein non-palmitoylated, while the L115R mutation caused almost all proteins to remain non-palmitoylated (Figures 3B,D,E). The palmitoylation ratio (palmitoylated over non-palmitoylated CSP α) decreased sharply for L115R mutation from around 2.23 to 0.18, and the palmitoylation score for C128Y mutation changed to 0.466 (Figures 3B,G). Meanwhile, L115R mutation triggered the formation of SDS-resistant aggregates of > 180 kDa, accompanied with a relatively lower quantity of endogenous CSP α (Figures 3B,C,F), indicating the aggregates assembling the endogenous proteins. This novel C128Y mutation induced a relatively smaller portion of aggregate formation from the exogenous proteins, with almost no change to the number of endogenous proteins (Figures 3B,C,F).

### C128Y Mutation Induced Lipofuscin Deposit in Granule Cells *in vivo*

**FIGURE 4:**
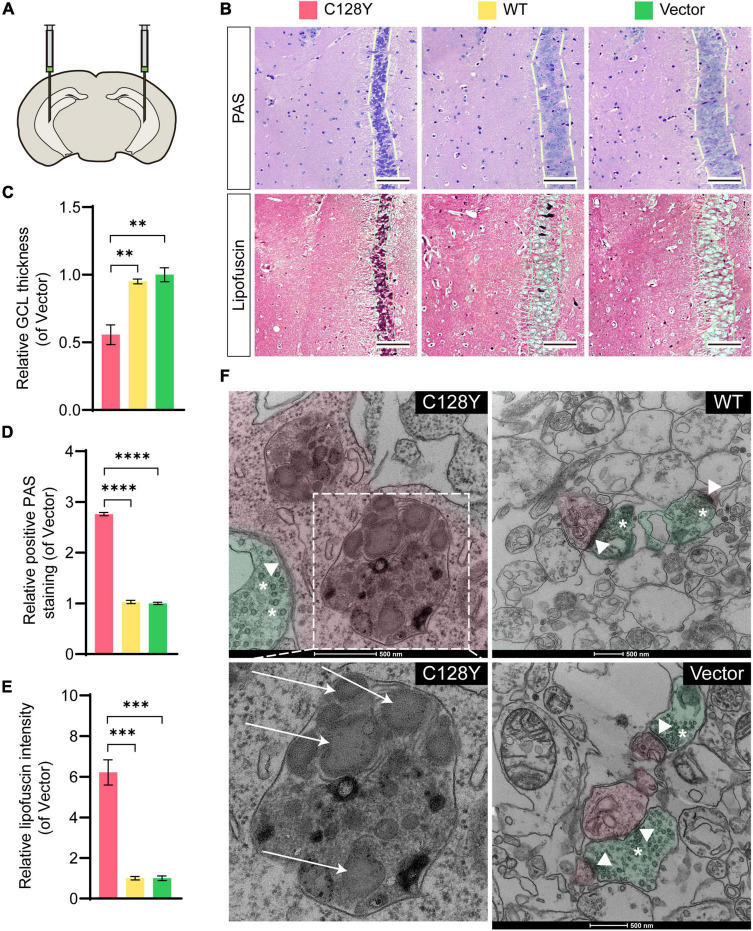
Aberrant granule cell layer and PSDs as a result of the C128Y mutation *in vitro.* **(A)** Bilateral hippocampal CA3 was delivered with lentivirus to overexpress mutant or wild-type CSP α. **(B)** Representative pictures show the granule cell layer of the mutant-injected hippocampal CA3 region is thinner as quantified in panel **C**, and with PAS-positive storage material in the granule cell perikarya (quantified in panel **D**), which was verified to be lipofuscin by the long Ziehl-Neelsen staining (quantified in panel **E**). **(F)** A representative picture showed the ultrastructure of the hippocampal CA3 region, with pseudo-green used to highlight the structure of a presynaptic neuron, and with pseudo-red demonstrating a postsynaptic neuron. C128Y mutation induced the enlargement of the lysosome compartment filled with heterogeneous contents, mainly the GRODs (white arrow), and the PSD (arrowheads) decreased remarkably. *N* = 3, all data in the figure are shown as mean ± SEM, each group vs. vector. *P < 0.05, ^**^P < 0.01, ^***^P < 0.001, and ^****^P < 0.0001. White arrows: GRODs; arrowheads: PSD; asterisks: synaptic vesicles. GRODs, granular osmiophilic deposits; PSD, postsynaptic density.

**FIGURE 5:**
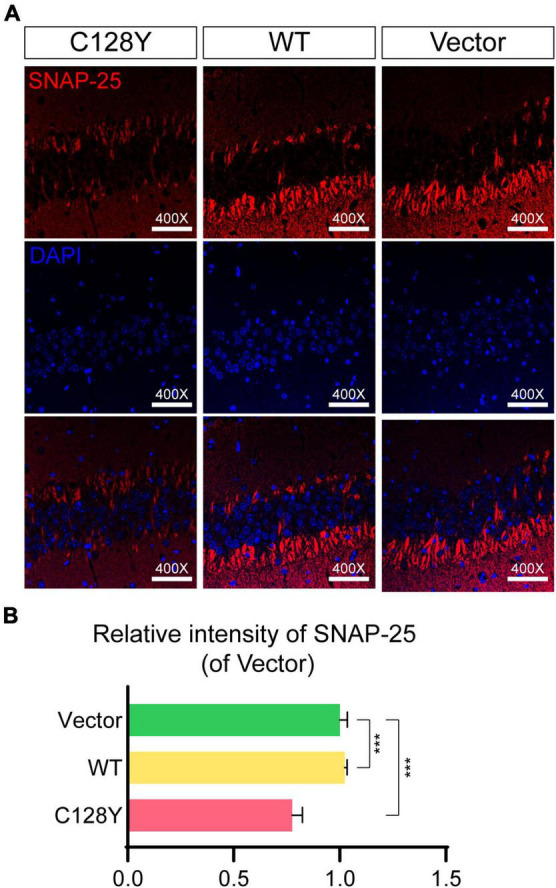
Changes of SNAP-25 expression in hippocampal CA3 region due to C128Y mutation. **(A)** Representative pictures show the decrease of SNAP-25 in the hippocampal CA3 region of mice expressing C128Y mutant CSP α, as quantified in panel **B**. *N* = 3, all data in the figure are shown as mean ± SEM, each group vs. vector. *P < 0.05, **P < 0.01, ***P < 0.001, and ****P < 0.0001.

We manually delivered lentivirus to the hippocampal CA3 region to observe both histological and ultrastructural changes due to mutant CSP α *in vivo* (Figure 4A). The efficiency of protein expression for lentivirus was similar in each group (Supplementary Figure 1). During daily feeding, mice neither showed the over-reaction to daily activities (changing cages or food/water replacement) or irritation phenomenon, nor seizures-like attack for mice expressing C128Y mutant proteins. Two months after injection, C128Y mutation induced granule cell atrophy and cell body became polygon-shape from oval- and water drop-shape, together with the decrease of granule cell layer thickness (Figures 4B,C). Light microscopy confirmed the deposit of glycogen-like material (positive by PAS staining) in the granule cell perikarya (Figures 4B,D). We further performed the long Ziehl-Neelsen staining (a method for detecting lipofuscin) on the brain slides, which verified PAS-positive storage material to be the lipofuscin (Figures 4B,E). Using TEM, we found that C128Y mutation caused the enlargement of the lysosome lumen in the postsynaptic neurons. These enlarged lysosomes were full of heterogeneous contents, mainly GRODs. Meanwhile, postsynaptic densities (PSDs) remarkably decreased in the mouse brain with C128Y mutant CSP α expression (Figure 4F). Decreased expression of SNAP-25, one marker of the synaptic compartment in the hippocampal CA3 region (Figure 5), was consistent with the above-mentioned alteration.

## Discussion

Previously, there have been reports about the L115R and L116Δ mutations in ANCL pedigrees (Naseri et al., 2021), and these mutations exhibit a manner of autosomal dominant inheritance. In the present study, we find the C128Y mutation in the female proband rather than her parents, suggesting that it might be a spontaneous point mutation.

Interestingly, this novel C128Y mutation resulted in different clinical manifestations compared to the L115R and L116Δ mutations. The initial symptoms for this patient were memory loss and personality change at the age of 20, whereas seizures were the most common chief complaints for previously reported cases in the mid- and late-20s (Naseri et al., 2021). Memory loss can precede the onset of the seizure, and in many patients, abnormal signals in electroencephalogram (EEG) are captured before clinical symptoms initiation. The EEG was normal for this patient until the recent follow-up. The patient developed the symptoms of parkinsonism at a clinically early stage, including tremor, bradykinesia, and an increase in muscle tension. These symptoms of parkinsonism have never been reported in patients with ANCL. From all the reported associated cases, parkinsonism was reported in ANCL type B and was associated with mutations in the *CTSF* gene (Berkovic et al., 2019). However, no mutation of *the CSTF* gene was detected in the proband and either of her parents. Another remarkable finding in the patient was the symmetric cortical atrophy and mild-moderate medial temporal lobe atrophy reported by the brain MRI, while the brain structure was reported normal by CT scanning at the first visit about 9 months earlier. Regarding the previously reported cases, the brain structure was reported normal by MRI or CT scanning for a long time after disease onset (Naseri et al., 2021). These different phenotypes seem to result from different mutation sites, as different amino acid harbors different biological effects and are involved in distinct mechanisms. Likewise, evidence from *in silico* analysis preliminarily supported that the novel C128Y mutation induced a harmful biological effect to organisms in a different pattern with the previously reported L115R mutation as is shown in Figure 2.

Among five domains of CSP α, both the DNAJ domain and cysteine-string domain are the key part of its function. Recent evidence shows that CSP α is important for neurogenesis as a complete deletion in mice could result in exhausting neural stem cells in the hippocampus, which could be ameliorated by blocking the mTOR pathway (Nieto-González et al., 2019). Considering the cell viability, both C128Y and L115R mutant proteins had no direct effects. The influence of CSP α mutation on the PI3K/AKT/mTOR pathway still needs further investigation.

To our knowledge, only a few pathogenetic mutations in the *DNAJC5* gene, including L115R (leucine-arginine substitution), L116Δ (a deletion of leucine), and C124_C133dup mutation (30 bp duplication), were identified, precisely locating at the whole cysteine-string domain. Here, we identified a novel variant in the genomic sequence of *DNAJC5* in a young Chinese female, which resulted in the cysteine substitution still in the cysteine-string domain. The cysteine-string domain provides an abundant amount of palmitoylation site for reaction with palmitic acid, and palmitoylated CSP α is necessary for membrane targeting and works as co-chaperone (Greaves and Chamberlain, 2006; Greaves et al., 2008, 2012; Diez-Ardanuy et al., 2017). In this study, the overall amount of expressed protein was similar in each group. Both C128Y and L115R mutations caused aberrant protein palmitoylation and oligomer formation. However, L115R mutation exhibited the inhibition of palmitoylation and remarkable aggregates formation by recruiting endogenous CSP α (Imler et al., 2019). It is reported that iron-sulfur clusters play an important role in aggregates formation and would combine with mutant protein by the Fe-S cluster scaffolding protein (Naseri et al., 2020). Further study needs to testify the combination between Fe-S cluster scaffolding protein and the novel C128Y mutant protein.

Regarding the neuropathological findings, the typical ultrastructural findings for patients with ANCL are the GRODs, curvilinear profiles, and fingerprint profiles (Radke et al., 2015). The CSP α is only limitedly expressed in several cell types, which are neurons and neuroendocrine cells (The Human Brain Atlas: https://www.proteinatlas.org/). The eccrine sweat gland epithelial cell by skin biopsy was used as an alternative for the brain tissue to observe the structural changes in the proband, and the GRODs were observed. Similarly, the C128Y mutation triggered the GRODs formation in the postsynaptic neurons of the mouse brain (as in Figure 4F).

Interestingly, the hippocampal granule cells address a special role in learning and spatial memories (Szabadics et al., 2010; Vyas and Montgomery, 2016; Hainmueller and Bartos, 2018), and in the present study, we found wearing thin of granule cell layer and cell body atrophy in mouse brain expressing the mutant protein, except for lipofuscin deposit. Although granule cell dispersion has been linked to chronic epilepsy (Weninger et al., 2021), recent studies pointed out that such correlation might result from the sampling bias, tending to be a normal variation in people without epilepsy (Roy et al., 2020). Up to our recent follow-up, the EEG of the affected patient was still normal, and she did not develop a seizure-like attack. Likewise, we did not observe that the mouse overreacts to daily manual operation or any sight of aggressiveness to littermate. It must be admitted that we did not assess them by the behavior tests, for instance, analysis of fine motor (head nodding) and falling frequency, or EEG measurement, as a preliminary experiment in a mouse model with a limited sample size. A knock-in mouse model by CRISPR-Cas9 technology is further needed to unmask these puzzles (Faller et al., 2015).

The CSP α targets the organelle’s membrane and has a great impact on neurotransmitter release from vesicles and organelle biogenesis by mediating membrane fusion (Ballabio and Bonifacino, 2020). Meanwhile, CSP α mostly locates at neurons and interacts with SNAP-25 and SNAREs (soluble, N-ethylmaleimide-sensitive attachment receptors) (Gundersen, 2020). The PSD contains a wide variety of hundreds of proteins and is an EM-electron dense region localized at the postsynaptic sites. PSDs are essential for synaptic integrity and plasticity and their decrease stands for a sign of neurodegeneration (Gorenberg and Chandra, 2017; Dejanovic et al., 2018). Neuron loss and synaptic dysfunction have been identified by the brain biopsy for a patient with AD-ANCL (Benitez et al., 2015). We found the decrease of PSDs in the mouse brain expressing mutant CSP α, potentially by decreasing the SNAP-25, which was similar for the L115R mutation (Naseri et al., 2020). The PSDs should be assessed in the brain of patients with ANCL carrying L115R or L116Δ mutation to verify its postmortem diagnostic value for ANCL. In the future, we need to further investigate the mechanism of C128Y mutant CSP α on neurodegeneration.

## Conclusion

In the present case, we reported a novel C128Y variant in the CSP α in a young Chinese female with ANCL, who presented with prominent memory loss, personality change, and parkinsonism together with brain atrophy by neuroimaging. Next, this study using cellular models demonstrates that the mutant CSP α exhibits aberrant protein palmitoylation and oligomer formation in a different pattern compared with the reported L115R mutant protein. *In vivo*, C128Y mutation triggers granule cell atrophy and decrease of granule cell layer thickness and PSDs in mice model, which provided some insights about some clinical manifestations that the patient presented. Further study needs to be performed to verify the mechanism of such biological effects for the C128Y mutation in detail.

## Data Availability Statement

The original contributions presented in the study are included in the article/Supplementary Material, further inquiries can be directed to the corresponding authors.

## Ethics Statement

The studies involving animal and human participants were reviewed and approved by Ethical Committee of Ruijin Hospital, Shanghai Jiao Tong University School of Medicine. The patients/participants provided their written informed consent to participate in this study.

## Author Contributions

QH, Y-BH, Y-FZ, R-JR, and GW designed the study. Y-BH, X-YX, RT, and R-JR performed the whole-genome sequencing-related work and clinical diagnosis. QH, Y-BH, Y-FZ, L-JL J-PL, J-TW, and X-QC performed the experiments and collected the data. QH, Y-BH, Y-FZ, ED, and GW analyzed the data. QH and GW wrote the manuscript. All authors contributed to the article and approved the submitted version.

## Conflict of Interest

The authors declare that the research was conducted in the absence of any commercial or financial relationships that could be construed as a potential conflict of interest.

## Publisher’s Note

All claims expressed in this article are solely those of the authors and do not necessarily represent those of their affiliated organizations, or those of the publisher, the editors and the reviewers. Any product that may be evaluated in this article, or claim that may be made by its manufacturer, is not guaranteed or endorsed by the publisher.

## Footnotes

1
http://www.ucl.ac.uk/ncl

2
https://www.ncbi.nlm.nih.gov/IEB/ToolBox/C_DOC/lxr/source/data/BLOSUM62

3
http://mutationtaster.org/

4
http://genetics.bwh.harvard.edu/pph2/

5
http://provean.jcvi.org/index.php

6
https://web.expasy.org/protscale/
