# Supplementary material for: Adult-Onset Neuronal Ceroid Lipofuscinosis With a Novel DNAJC5 Mutation Exhibits Aberrant Protein Palmitoylation
Source: Front Aging Neurosci. 2022 Apr 8;14:829573. doi: 10.3389/fnagi.2022.829573 (PMC9031920; doi:10.3389/fnagi.2022.829573)
Supplement: Supplementary file 1 [file Data_Sheet_1.docx]

1. **Clinical features of the proband**

A 20-year-old female undergraduate initially presented to her primary physician with subjective memory loss and personality changes reported by her parents. Then, the patient was referred to a neurologist, and at this visit, her cognition was assessed by the scales and showed no abnormality with her peers. At this visit, there were no remarkable findings in the neurological examination, together with no abnormality in brain structure reported by the Brain computed tomography (CT) scanning. Then, she discharged from the hospital without further treatment. The patient’s next evaluation was conducted at the memory clinic, Ruijin Hospital, Shanghai Jiao Tong university School of Medicine about 9 months later. The patient’s parents reported progressive cognitive decline since last departure, and now her daily activities were influenced severely. At this visit, the patient presented with parkinsonian motor features, including tremor in the left upper limb and bradykinesia. During the neurological examination, her facial expression relatively decreased and the muscle tension increased. She scored 16 out of 30 on the mini-mental state examination (MMSE), 11 out of 30 on the Boston naming test (BNT), 1 out of 4 on the Clock Drawing test (CDT), and 43 out of 100 on the Addenbrooke’s cognitive examination (ACE-R). Brain magnetic resonance imaging (MRI) revealed prominent atrophy of bilateral cerebral cortex (Global cortical atrophy (GCA) scale = 2) and hippocampus (Medial Temporal lobe Atrophy (MTA) scale= 2) (Figure 1B).

Routine laboratory tests including blood cell count, liver and kidney function panels, tests for human immunodeficiency virus (HIV) and syphilis, thyroid function, hormone panel, folate and vitamin panels showed no abnormalities. The autoimmune panel revealed slight elevation of anti-streptolysin (ASO) and a mild decreased level of C3, which, however, indicated fluctuation irrelevant to clinical symptoms. Both electromyography (EMG) and electroencephalogram (ECG) of this patient were normal. Tests of ceruloplasmin and Kayser-Fleischer (K-F) ring, blood and urine tandem mass spectrometry and Huntington’s disease (HD) CAG repeats were negative.

Whole-exome sequencing identified a mutation in *DNAJC5* exon4 (C.383G>A), which was further verified by PCR (Figure 1C). This novel C128Y mutation was only detected in the proband and not her parents. And mutation in all other disease-associated genes of NCL had not been detected. The results of other necessary examinations for differential diagnosis were listed in the supplementary of this article. The skin biopsy identified granular osmiophilic deposits (GRODs) ultrastructurally using transmission electron microscopy (Figure 1D). Taking all the clinical manifestations, neurological examination, skin biopsy and novel C128Y mutation in *DNAJC5* gene together, the patient was eventually diagnosed as ANCL.

1. **Table of primers for plasmid verification**

Table S1. Table of primers for plasmid verification

| CSP α WT | |
| --- | --- |
| Forward: | 5′-AAAAGAATTCATGGCAGACCAGAGACAGCGCTC-3′ |
| Backward: | 3′- CCCGCAGCAGCAGTTGAAGTAGCAGCACAG-5′ |
| CSP α L115R | |
| Forward: | 5′-TTCGTCTTCTGTGGCC(T/G)CCTCACCTGCTGCTAC-3′ |
| Backward: | 5′-GTAGCAGCAGGTGAGG(A/C)GGCCACAGAAGACGAA-3′ |
| CSP α C128Y | |
| Forward: | 5′-TGTCTGTGCTGCT(G/A)CTTCAACTGCTGCTGC-3′ |
| Backward: | 5′-GCAGCAGCAGTTGAAG(C/T) AGCAGCACAGACA-3′ |

1. **Fluorescence in situ hybridization in hippocampal CA3 region.**


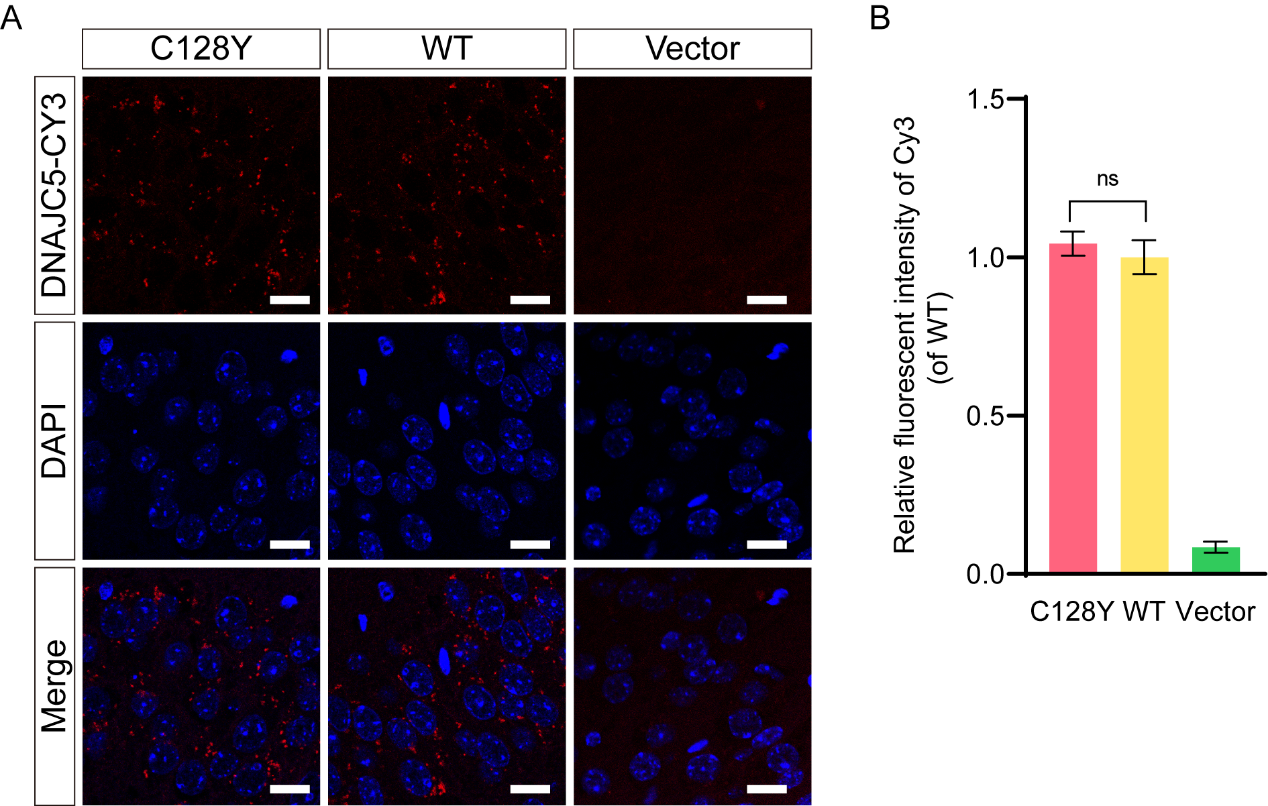


Figure S1. Protein expression level of CSP α in hippocampal CA3 region.

A. The protein expression level of CSP α was similar in the C128Y group and WT group verified by fluorescence in situ hybridization, as quantified in B.
